# Chordoma of the lumbar spine—A potential diagnosis not to be forgotten!

**DOI:** 10.1016/j.radcr.2022.10.037

**Published:** 2022-11-24

**Authors:** Rafik Elafram, Oussama Abcha, Majdi Ben Romdhane, Majdi Sghaier, Hedi Annabi

**Affiliations:** Interior Forces Hospital La Marsa, Tunis Manar University, Interior Forces Hospital La Marsa, Tunis, Tunisia

**Keywords:** Chordoma, Lumbar spine, Surgery

## Abstract

Chordoma is a rare, malignant neoplasm thought to develop from the notochord. It most commonly occurs in the base of the cranium or the sacro-coccygeal region but around 15%-20% affect the vertebral body. Extra-lesional resection with or without adjuvant radiotherapy is generally accepted as the mainstay of treatment for this slow-growing tumor. We present a case whereby a patient with an extensive vertebral body lesion causing caudal compression, treated with spinal decompression and posterior stabilization. This case highlights the importance of pre-operative tissue diagnosis, and that, although rare (0.8 per 100,000), chordoma should always be considered.

## Introduction

We present an extensive case of lumbar chordoma causing tail compression, initially thought to be spinal echinococcosis based on morbidity and radiological studies. Therefore, this patient underwent a 2-stage procedure, first with posterior stabilization of spinal decompression, at which point extralesional resection was more appropriate.

## Case report

**Fig. 1:**
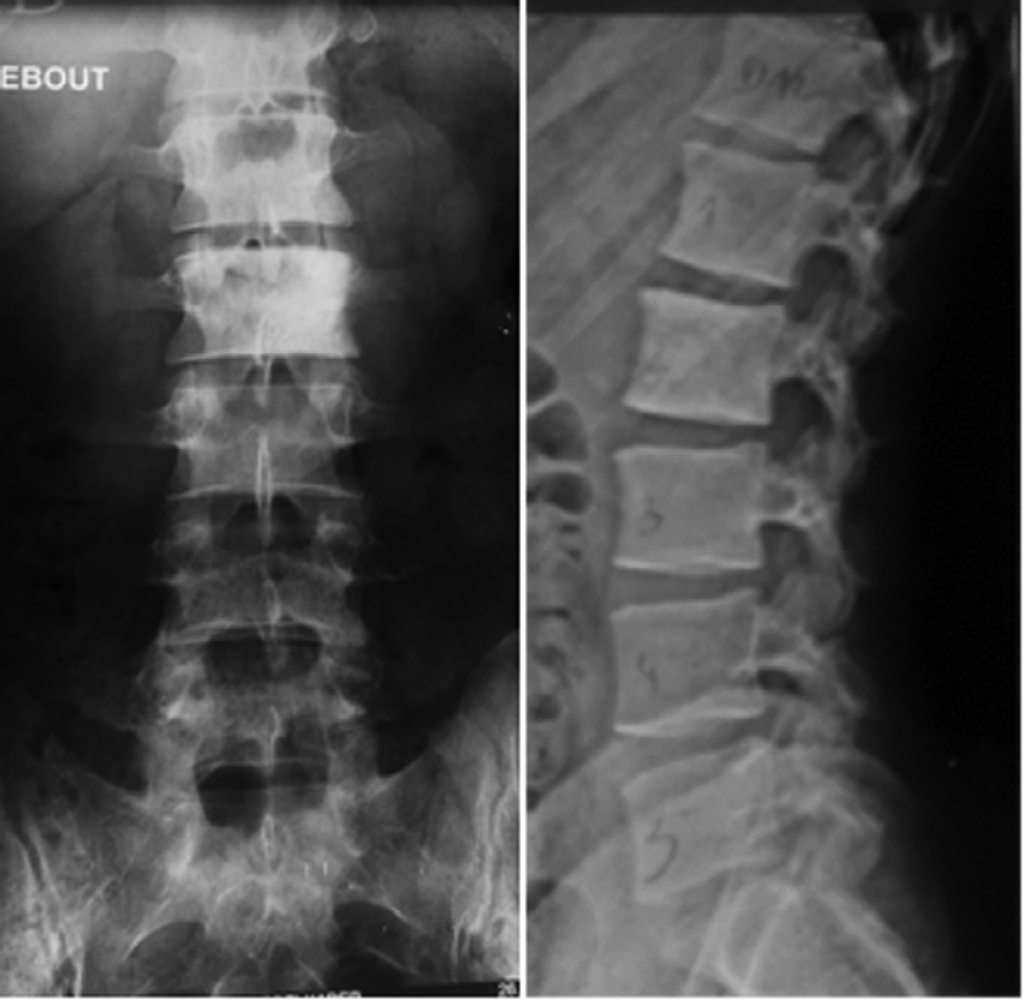
Radiograph of the lumbar spine illustrating sclerosis of L2 vertebral body and a destructive lesion of L1.

A 28-year-old trainer presented with a 6-month history of back pain and increased leg pain and weakness. (Foot numbness? Foot pain?). He reported no history of trauma. His history is unremarkable. Lumbar radiographs showed sclerotic L2 and destructive L1 vertebral bodies associated with osteolytic destruction of the L1-L2-L3 pedicles. There was no evidence of a paravertebral mass (Fig. 1).

**Fig. 2:**
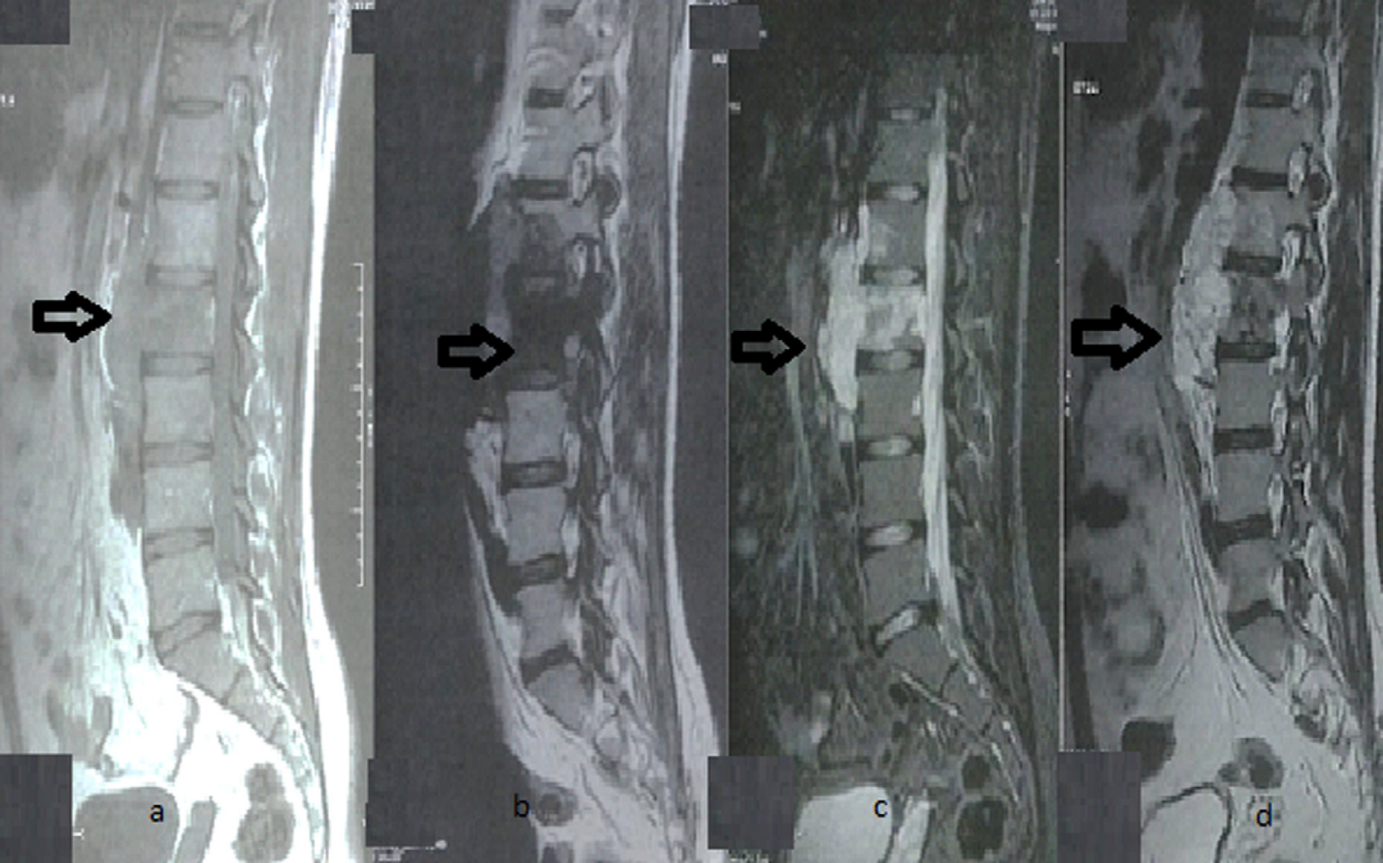
T1 (A , B) T2 (C, D)-weighted sagittal MRI of lumbar spine showing bone-based malignancy from L1 to L3.

**Fig. 3:**
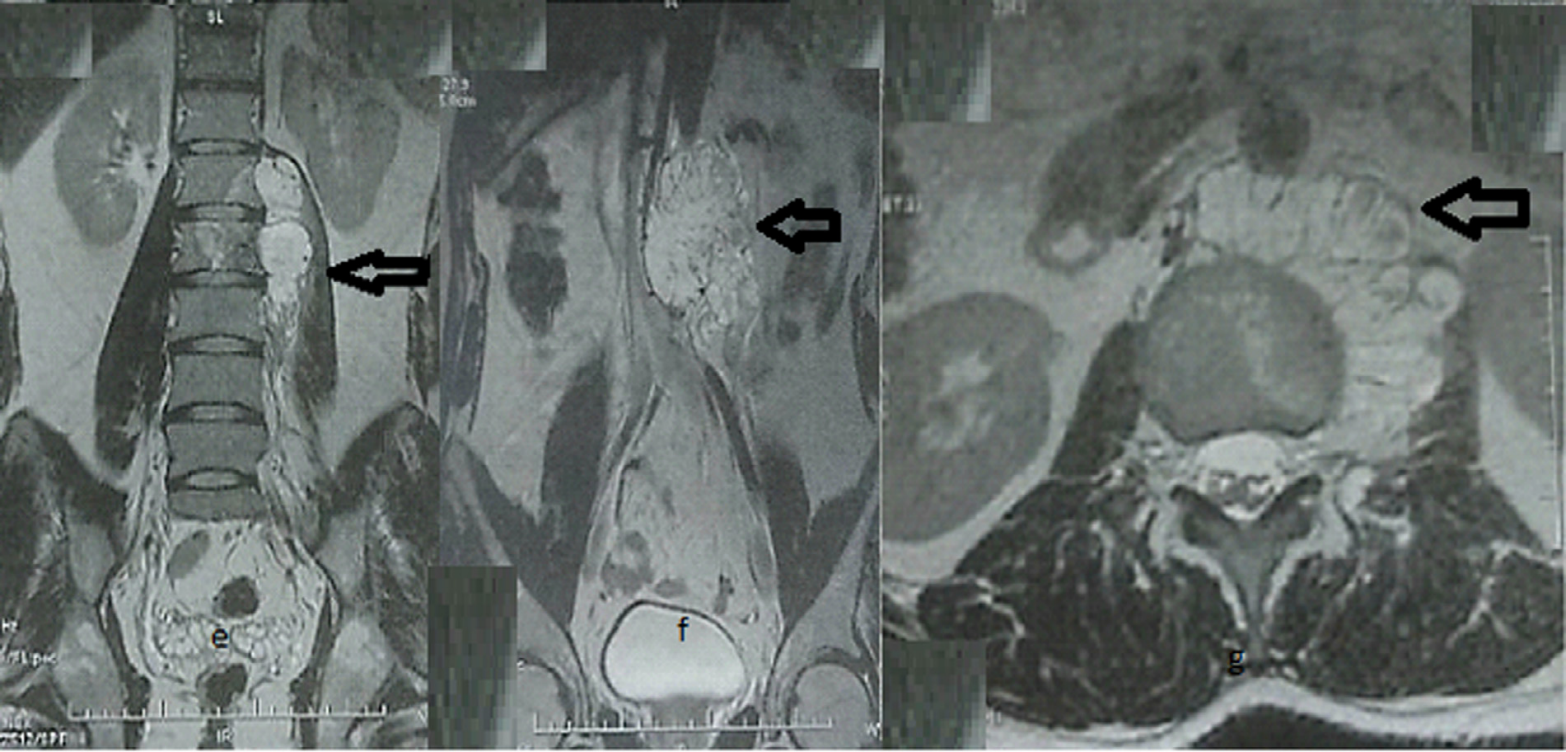
(E, F) T2-weighted coronal MRI of abdomen shows malignancy expanding into the abdomen and psoas muscle. (G) T2-weighted axial MRI of lumbar spine highlighting borders of tumor infiltrating posteriorly into the paraspinal musculature.

Magnetic resonance imaging (MRI) showed extensive heterogeneous bone-based malignancy in the paravertebral space extending from L1 to L3 with hypointense T1 signal and hyperintense T2 signal, without significant enhancement after gadolinium injection. It results in severe canal stenosis with posterior elements extending posteriorly to the left posterior arch of L3 and anteriorly to the psoas and abdomen (Figs. 2 and 3).

Since the patient was a dog handler and based on frequency and radiological studies, the most likely diagnosis was spinal hydatid disease.

### Operative treatment

**Fig. 4:**
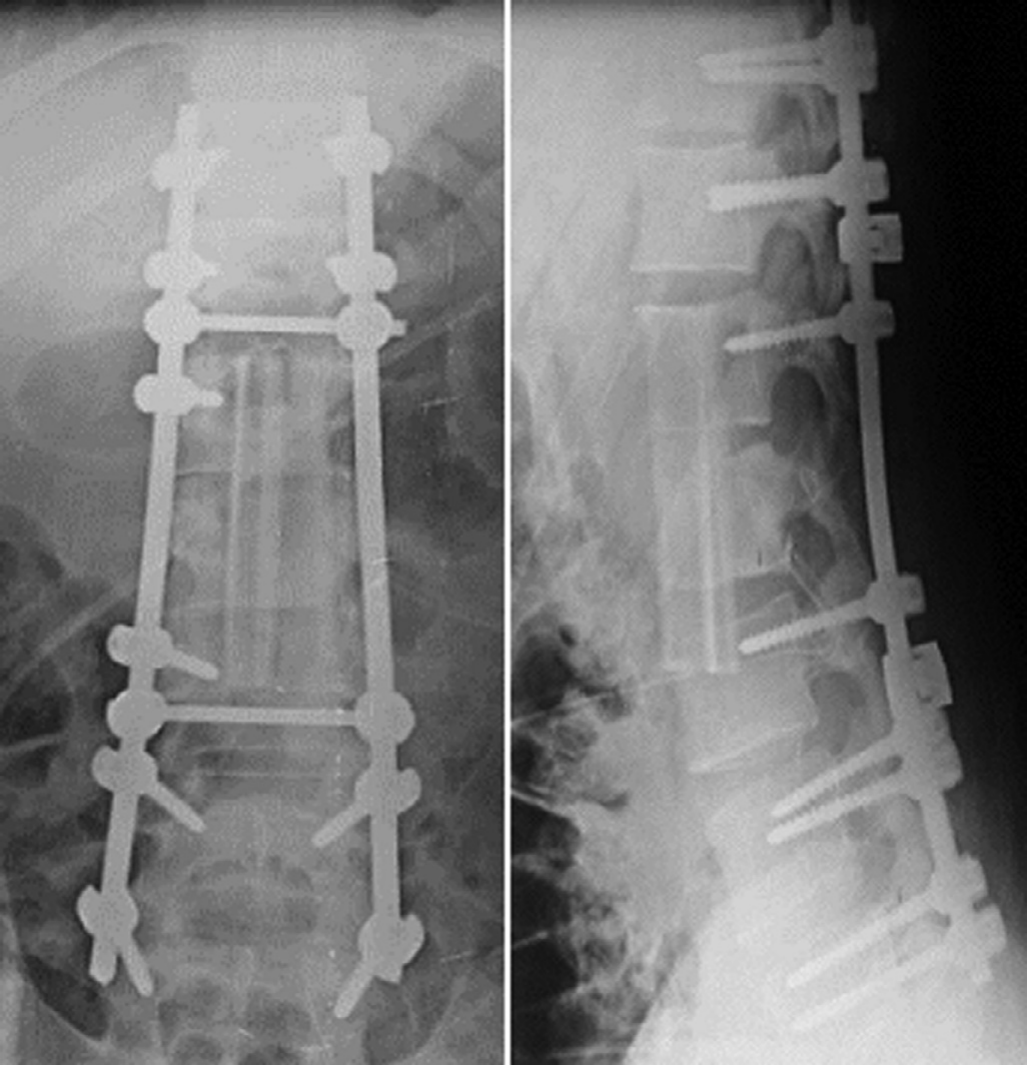
Proximal pedicle screw fixation from T12 to L2 and distal fixation from L4 to S1.

Due to the large size of the chordoma, a double-approach resection was planned. The first was a posterior approach consisting of a D11-L5 instrument and his L1-L2-L3 left hemilamine resection. An anterior approach was performed 12 weeks after his posterior approach. A multilobed tissue tumor was found at retroperitoneal exposure. Aspiration revealed vesicular structures potentially consistent with echinococcosis. Apsoas resection, L1-L2 hemisection, and partial resection of the L3 superior endplate were performed. The bone defect was reconstructed with a non-vascularized fibular graft from the left leg. The patient survived the procedure without any problems and received albendazole as adjuvant drug therapy. One year later, the patient was asymptomatic, disease-free, with an acceptable level of activity and a stable spine (Fig. 4).

**Fig. 5:**
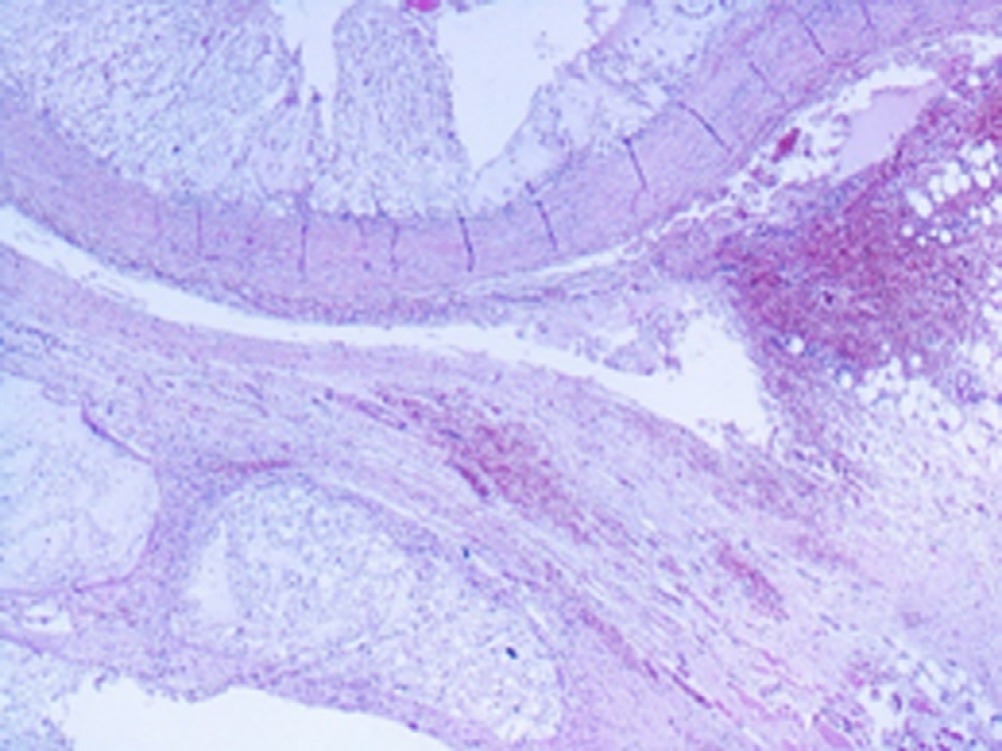
Chordoma tumor nodules and adjacent skeletal muscle. Hematoxylin and eosin stain and magnification ×20.

### Pathological diagnosis

**Fig. 6:**
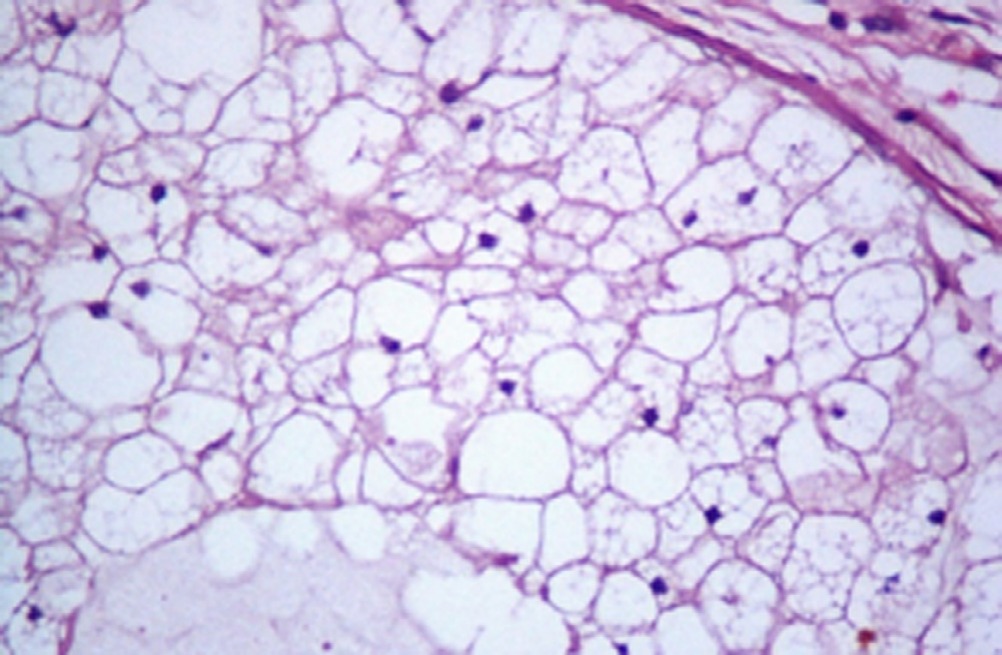
Characteristic chordoma histomorphology of large, pale, vacuolated physaliphorous tumor cells in amongst myxoid stroma. Hematoxylin and eosin stain and magnification ×20.

**Fig. 7:**
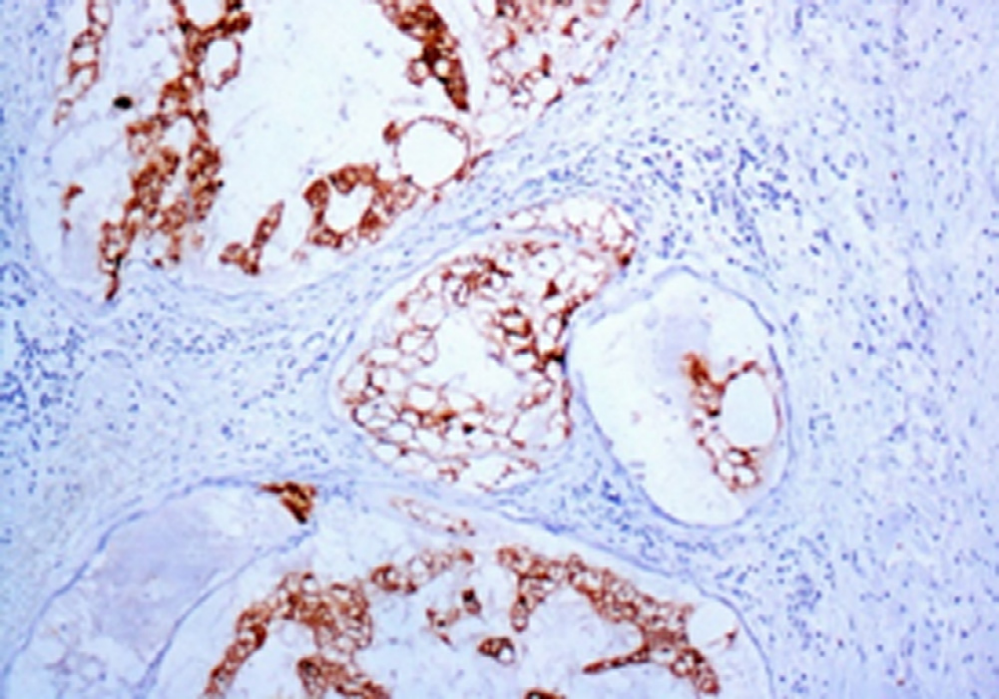
Pan-cytokeratin immunohistochemistry highlighting tumor cells. Magnification ×10.

Histopathological inspection of the resected mass revealed multiple varying sized nodules of large, pale, multivacuolated physaliphorous cells showing mild nuclear pleomorphism, with some areas of metaplastic cartilage formation within the tumor. Immunohistochemistry was positive for CK, PS100, vimentine and EMA staining (C68 negative) (Fig. 5). Histological features in conjunction with immunohistochemical profile were consistent with the diagnosis of chordoma (Figs. 6 and 7). The patient did not have spinal hydatid disease.

## Discussion

Chordoma is a primary bone tumor that arises from remnants of the notochord is characterized by slow growth over years and often spreads to the lungs, bones, soft tissues, liver, skin, or lymph nodes. It is associated with late metastases in 40%-60% [1,2].The 5-year survival rate (5YSR)is 50% and the 10-year survival rate(10YSR)is 28% [3]. The location and size of the chordoma have a significant impact on the outcome. Chordomas originating from the spine are more likely to be locally aggressive and metastatic than other chordomas (80% vs 43%) and have a lower5YSR.Lumbarchordoma,such as ours, has a higher rate of metastasis than any other site [[2], [3], [4], [5]], but local aggressiveness is more likely to cause death and disability than metastasis [3]. Most affect the base of the skull and the sacrococcygeal region, with about15%-20% affecting the vertebral bodies. Signs and symptoms often appear to be the result of discogenic or nonspecific pathology, leading to delayed diagnosis [4].

Symptoms most commonly result from compression of the anterior columns and roots. The most common complaints of patients with lumbar chordoma are pain and paresthesia. Motor changes can occur, but paraplegia is rare [2]. Our patient had been complaining of back pain for 6months.Preoperativediagnosis of the lumbar chordoma is also difficult because it is often confused with the more common tumors of the lumbar spine, such as giant cell tumors, myeloma, metastasis, hemangiomas, and hydatid myeloma [2]. MRI is currently the imaging technique of choice for preoperative diagnosis and evaluation. MRI can detect small chordomas before larger symptoms develop, improving patient prognosis and increasing the likelihood of tumor resection [6].Chordoma appears hypointense or isointense on T1-weighted images, but hyperintense on T2-weighted images [2]. On radiographs, the vertebrae appear irregular because the chordoma grows slowly and allows bone remodeling. Chordomas may bilobed and soft or hard with focal areas of necrosis, cysts, ossification, hemorrhages, and calcifications. Characteristically, their histological appearance includes vacuolated cells with mucus-forming physaliphores. Surgical resection has become more aggressive over time, evolving from lesion reduction to complete resection, aiming for complete tumor resection with wide margins, decompression, reconstruction, and stabilization [2]. Aggressive excision is the only curative treatment for the following reasons:

1. Clinical symptoms are generally delayed. In other words, chordoma is often large at diagnosis and other oncological treatments are inappropriate [2,3,6].
2. Poor surgical margins are associated with poor survival and a high recurrence rates
3. Local tumor progression is a strong prognostic factor that reduces survival [4,5].
4. Chordoma is resistant to conventional radiotherapy (cured only at toxic doses [60 Gy]) and chemotherapy [1].

However, in most scenarios, marginless resection is difficult due to the surrounding neurovasculature and extensive tumor size [4,5]. Radiation therapy continues to serve as a recurrence prevention for patients with inadequate surgical resection and intralesional/narrow margins, and as a definitive treatment for inoperable patients [4,7]. This role may increase with the advent of stereotactic radiotherapy. Although chordoma is generally chemo-resistant, imatinib mesylate has shown promise in clinical trials [1,7], with a prospective, multicenter phase II study of 55 patients showing a clinical benefit of 73% at 1 year has been shown [4]. Although recent developments in radiotherapy and chemotherapy are promising, controlled long-term follow-up studies to guide future practice are lacking [6]. Unfortunately, en bloc resection could not be performed due to technical limitations. The large size of our patient's chordoma prompted an aggressive, staged complete surgical excision of the tumor with planned violation, using a fibular graft for reconstruction.

En bloc resection can improve survival and reduce the rate of local recurrence, and is often used to treat chordoma [6]. En bloc resection is the complete removal of the tumor as a whole. Theoretically, a complete en bloc resection ensures no residual tumor, thereby preventing tumor cell contamination. Boriani et al. [6] a review of studies clearly shows that unrelated en bloc resection is the only treatment that achieves disease-free status. En bloc vertebral resection can increase disease-free 5YSR from 50% to 70% up to 100% by reducing the risk of recurrence [7], but lesions with or without radiotherapy Slow-growing local recurrence is inevitable after internal or en bloc surgery. Resection of the lesion. Recurrence occurs on average 26 months after treatment. Despite some longevity (up to 10 years), the pain/disability caused by tumor regrowth reduces quality of life [6].

In our case, the patient underwent a combination of anterior and posterior L1-L2 stage hemirectomy and partial resection of the L3 upper endplate. No studies have compared the effectiveness of the combined posterior and anterior-posterior approaches. Theoretically, a posterior approach alone is inadequate for direct visualization of abdominal structures and increases the likelihood of major vascular injury. The combined approach has the disadvantage of longer operative time for patients, but it can be done alternately or simultaneously. Due to the extensive nature of our patient's chordoma, a stepwise approach was most appropriate. The overall complication rate after total en bloc resection is 36.3% in most studies. Complications include severe neurovascular injury, excessive bleeding, tumor cell contamination, spinal instability, and abdominal herniation (after anterior approach) [6,7].Resection of spinal tumors results in significant loss of posterior elements and biomechanical instability, making bone reconstruction and arthrodesis essential [5,8]. Although there are many well-studied options for anterior reconstruction, options for successful long-term posterior reconstruction are still in their infancy. Instrumented posterior reconstructions often fail in the long term because biomechanical forces induce fatigue. Biological reconstruction with posterior arthrodesis and anterior brace circumvents this problem, but adjuvant chemotherapy/radiation poses a problem because it impairs healing and increases the likelihood of arthrodesis failure [8]. For this reason, we have to resort to adjuvant radiotherapy.

## Conclusion

Lumbar chordomas are rare, with long-standing symptoms prior to diagnosis. A high suspicion rate should be present when dealing with a patient who shows pathological nonspecific image studies. MRI images though not pathognomonic, may help reduce diagnostic delay. CT-guided biopsy, with immunohistochemical sample studies, should be thoroughly performed. Treatment can be potentially curative only if aggressive surgical resection is utilized.

## Patient consent

The authors declare that they have the patient consent.

## References

[1] Jawad MU, Scully SP (2010). Surgery significantly improves survival in patients with chordoma. Spine.

[2] Tuna H, Aydin V, Bozkurt M, Attar A (2005). Chordoma of the lumbar spine: a case report. Neurocirugia.

[3] Hsu KY, Zucherman JF, Mortensen N, Johnston JO, Gartland J (2000). Follow-up evaluation of resected lumbar vertebral chordoma over 11 years: a case report. Spine.

[4] Ferraresi V, Nuzzo C, Zoccali C, Marandino F, Vidiri A, Salducca N, Zeuli M, Giannerelli D, Cognetti F, Biagini R (2010). Chordoma: clinical characteristics, management and prognosis of a case series of 25 patients. BMC Cancer.

[5] Jung Y, Shin H (2009). Combined anterior and posterior en bloc vertebrectomy for lumbar chordoma—case report. J Korean Nuerosurg Soc.

[6] Boriani S, Bandiera S, Biagini R, Bacchini P, Boriani L, Cappuccio M (2006). Chordoma of the mobile spine: fifty years of experience. Spine.

[7] Cloyd J, Acosta F, Polley M, Ames C (2010). En bloc resection for primary and metastatic tumours of the spine: a systemic review of the literature. Neurosurgery.

[8] Eastlack R, Dekutoski M, Bishop A, Moran S, Shin A (2007). Vascularised pedicled rib graft: a technique for posterior placement in spinal reconstruction. J Spinal Disord Tech.

